# Supplementary material for: Are carbon emissions trading and green financial instruments synergistic? -Comprehensive quantitative research based on content analysis
Source: PLoS One. 2024 Mar 7;19(3):e0298601. doi: 10.1371/journal.pone.0298601 (PMC10919601; doi:10.1371/journal.pone.0298601)
Supplement: S1 Data — (ZIP) [file pone.0298601.s001.zip › Data/地级市Z_F工作报告.zip!/╜¡╬≈/╕o╓▌╩╨/└┤╫╘╣1⁄2╓┌║┼í╛┬φ┐╦╩2╛▌═°í┐_2019.docx]

各位代表：

现在，我代表区人民政府向大会报告工作，请予审议，并请区政协委员和列席会议的同志提出意见。

一、2018年工作回顾

2018年，在市委、市政府和区委的坚强领导下，全区上下认真学习贯彻习近平新时代中国特色社会主义思想和党的十九大精神，从更高层次贯彻落实习近平总书记对江西工作的重要要求，紧紧围绕省委、省政府“创新引领、改革攻坚、开放提升、绿色崛起、担当实干、兴赣富民”的工作方针和市委、市政府“坚持一个指导、突出三项攻坚、打好四大升级战”的工作思路，大力实施“转作风、做民事、促稳定、保发展”十二字工作总部署，坚持稳中求进工作总基调，贯彻新发展理念，落实高质量发展要求，经济社会实现总体平稳、稳中有进、稳中提质的发展态势。初步核算，全年实现生产总值457.2亿元，增长8.8%；财政总收入23.66亿元，增长4.6%，其中税收占比为87.5%，比2017年提高2.3个百分点，再创历史新高；固定资产投资增长12%；规模以上工业增加值增长9.1%；社会消费品零售总额215.1亿元，增长10.5%；实际利用外资5220万美元，增长11%；外贸出口15.7亿元，增长1%；城镇居民和农村居民人均可支配收入分别达到38850元和18000元，分别增长8.5%和9%。

这一年是狠抓产业升级、促进实体经济振兴的一年。

工业经济硕果累累。全年实现规模以上工业企业主营业务收入180亿元，增长11.2%；工业税收7.4亿元，增长26.8%；工业用电总量6.6亿千瓦时，增长15.8%；新增规模以上工业企业10家。与此同时，重新擦亮临川“建筑之乡”的金字招牌，建筑施工企业完成建安总产值230亿元，增长12%；实现本地建筑业税收3.8亿元，增长44%。一是培育新动能。着力推动电子信息、食品加工、生物医药等主导产业集聚，有效改变我区工业过度依赖有色金属加工产业的短板。特别是电子信息、食品加工产业茁壮成长。高质量建设电子科技产业园，已入驻联顺半导体、华显智能、世星科技等7个生产项目，其中5家企业春节前可试投产。大健康食品产业园一期11万平方米厂房拔地而起，设备已进场安装，春节后可试投产。二是建设新速度。在抚北工业园区进一步加大闲置低效工业用地的清理力度，通过整合、嫁接、重组等方式盘活企业8家、用地640亩。在才都工业区重点推进电子科技产业园、大健康食品产业园建设，累计建设标准厂房60余万平方米。污水处理厂、110KV变电站和园区路网等基础设施日益完善，才都工业区项目承载力得到进一步增强。三是打造新环境。按照“四最”环境要求，全面贯彻落实省、市“降成本、优环境”系列优惠政策，落实企业政策性退税1.85亿元。着力打造好信息、融资、技术、用工四大服务平台，在生产要素保障、政策扶持等方面给予倾斜，千方百计促进企业健康发展。充分发挥“财政过桥资金”和“财园信贷通”作用，帮助企业解决资金3.86亿元，有效缓解企业融资难题。

现代农业跃然升华。大力实施乡村振兴战略，被评为全省农业农村工作综合先进县(区)。一是点上有突破。全区现有农业产业化龙头企业67家，其中省级龙头企业达到18家；发展农民专业合作社1091家，其中国家级示范社5家、省级示范社14家，农户入社率达到70%。发放财政“惠农信贷通”资金1.8亿元，为新型经营主体发展壮大、农业产业升级、农民增收致富增添强劲动力。二是线上有延伸。现代农业示范园及设施农业建设稳居全市前列，一批重大农业项目正按照计划有序推进，建成后将极大提升全区现代农业发展水平。昌抚态何源田园综合体一期工程已经完工，生态循环经济、稻田净水套养、热带水果北移种植等多项现代农业技术走在全市前列。华宸农业蔬菜科技产业园建成智能温室大棚6万平方米、生产大棚10万平方米，种植露天蔬菜西兰花1000亩，已成功创建省级现代农业示范园。昌抚牡丹田园综合体游客接待中心主体工程已竣工，已栽种牡丹20万株，将成为全省规模最大的牡丹观光基地。三是面上有拓展。全区已形成优质粮食、中药材、蔬菜、畜禽、水产、瓜果、花卉等七大特色产业，产值达58亿元，占全区农业总产值79%。2017年8.13万亩高标准农田建设已全面通过省、市、区三级验收；2018年11.46万亩高标准农田建设正在稳步推进。全区“三品一标”总数达122个。

新经济态势良好。一是旅游强区深入推进。大力发展全域旅游，依托山水禀赋和文化资源优势，描绘出“风景临川”的秀美画卷。灵谷峰景区成功创评国家4A级景区，实现我区零的突破；仙盖山农业示范园成功创评江西省5A级乡村旅游示范点，实现全市零的突破。成立“梦之旅”旅游公司，推出4条市内旅游精品路线，开通2条旅游公交线路直达景区。高质量建成民宿100套，让游客“玩在景区、住在乡村、吃在农家”。全区旅游接待人数730.2万人次，增长29.8%；实现旅游综合收入78.3亿元，增长29.9%。二是电子商务势头强劲。已有23个乡镇建立农村电商服务中心，200多个行政村建立村级电商网点，上线临川本地特色农产品达到100多种，培训带动新型经营主体和农村创业人员3000余人。2018年农村电商实现销售收入11亿元、增长22%，临川西瓜、葛粉、菜梗、虎奶菇、茶油等特色农产品均实现网上交易。三是健康养老业加速发展。鼓励社会力量兴办医养结合机构，将临川第三医院转型成为一所二级医养结合医疗机构。积极探索公办养老机构公建民营模式，区社会福利中心老年公寓整体公开拍租，引入湖南普亲公司进行市场化运营。大力发展居家养老服务，探索开展智能健康管理、医养结合等“互联网+”居家健康智慧养老服务。

这一年是狠抓改革开放、促进社会活力迸发的一年。

改革步伐更加坚定。扎实推进“放管服”改革，取消、调整行政权力事项132项，梳理“一次不跑”事项96项，大部分企业通过网络提交企业年报；梳理“只跑一次”事项182项，将企业办理注册登记压缩为1个工作日，材料齐全当场办结，实现企业注册开办3个工作日完成。对没有法律法规规定的证明事项一律取消，共计取消证明事项133项，确保“减证便民”行为取得实效。积极稳妥推进机构改革，临川区税务局已挂牌成立，展坪乡成功获批撤乡设镇。稳步推进殡葬改革，倡导文明节俭的丧葬理念，树立良好的社会文明新风尚。持续深化农业供给侧结构性改革，扎实推进农村土地“三权分置”改革、农村集体产权制度改革试点和农村宅基地管理试点。在全省率先开展农业综合水价改革、经济发达镇(唱凯镇)行政管理体制改革、农业大灾保险等试点工作，总结推广临川供销社基层组织建设、智慧百乡千村健康医养扶贫工程、公办养老机构公建民营模式的先进做法，为全省乃至全国提供“临川经验”。

争项争资更加显著。一方面，招商引资成效显著。重新组建4支专业驻外招商小分队，各由一名县级领导带队蹲点长三角、珠三角、闽三角等地开展产业招商，采取“轻资产、重资本”的招商模式精准发力。全区共签约引进项目65个，其中20亿元以上项目5个，亿元以上项目32个；实际进资达63亿元，增长14.5%。招商项目数量和实际进资均居全市前列，项目的建成必将为我区经济高质量发展注入强劲动力。另一方面，跑项争资成效显著。全力以赴争取国家、省、市对我区的支持，高起点、高标准、高质量地谋划一批既符合国家投资政策，又对我区经济发展有支撑引领作用的重大项目，全年累计向上争取各类资金21.14亿元。

开放平台更加开阔。一方面，积极融入南昌。在“南昌大都市圈”建设上下功夫，重点推进昌抚合作示范区建设。围绕“云山慢城”的功能定位，重点推进昌抚态何源田园综合体、昌抚家和特色小镇、昌抚牡丹田园综合体等产业项目。围绕完善基础设施稳步推进核心区供水工程、区间道路工程、客运站、云山110KV输变电工程、新增福银高速昌抚合作示范区互通等项目前期工作。天然气管网工程、10KV长云线工程等基础设施项目已完成。昌抚合作示范区从建设之初的“一张白纸”徐徐展开美丽画卷，成为引领抚州对接融入南昌大都市圈的“桥头堡”、“后花园”。另一方面，积极对外开放。新增外贸出口实绩企业4家，注册外贸自营出口企业3家，外贸出口结构呈现多元化趋势。中德体育、中德服饰率先在全市开行抚州—汉堡中欧班列，创造“可复制、可推广”的外贸出口货运模式，为全市企业参与“一带一路”积累了宝贵经验。

“双返双创”更加活跃。积极搭建创业平台、整合创业资金、树立创业典型，动员在外企业家返乡创新创业，引导其发挥自身资源优势，把新技术、新产品、新理念带回家乡。全年共新登记注册各类企业2014户，增长16%。新开业个体工商户6241户，增长18%。深入实施创新驱动发展战略，圆满通过江西省知识产权富民强县示范县(区)建设专项验收，11家企业通过2018年高新技术企业认定，自立环保和银涛药业成功入选国家知识产权优势企业。专利申请量达1350件，发明专利申请量达180件，专利授权量达1300件，均居全省前列。

这一年是狠抓项目建设、促进城乡协调发展的一年。

项目建设实现新突破。坚持“项目为王”的理念，2018年全区重点建设项目开工52个，完成投资101.26亿元。鼓励民间资本参与城区基础设施、生态文明建设、民生社会事业等重点项目建设，全年共实施PPP项目13个，吸引社会资本投资21.5亿元。正式启动群众盼望多年的“一河两岸”提升改造工程，总长7.1公里的路网工程基本完工，高品位打造了“一河两岸”景观提升、才子大桥、临汝书院、才子巷商业街等一批融入临川文化的重点项目。与此同时，加快和市中心城区融合步伐，建设南路东延伸段、学成路等一批连接市中心城区的道路已竣工。

秀美乡村呈现新气象。坚持把秀美乡村建设与乡村休闲旅游、脱贫攻坚、中药材种植和农村精神文明建设相结合，按照连线成片推进新农村建设要求，全面完成545个新农村村点建设任务。重点对部分316国道沿线进行环境提升，对云山、罗针等2个集镇进行提升改造。加快农村交通基础设施建设，完成285条通25户以上自然村公路的修建和一批区、乡道升级改造工作任务。积极推进乡村移风易俗，“道德红黑榜”覆盖全区所有社区和行政村，倡导丧事简办、喜事小办，促进乡风民风美起来。

环境卫生得到新提升。以“三城同创”为抓手，大力开展城乡环境综合整治行动，强力推进“门前三包”管理责任制，持续推进“五车”专项整治，全面实行城乡环卫一体化市场运作化管理模式，城乡环境不断改善。与此同时，农村拆临拆违工作全面铺开，799个自然村验收合格，拆除面积达160万平方米。交通主干道的铁皮房、铁皮顶已基本拆除，高标准房屋立面改造工作全面完成，全区农村环境基本实现“四无、四净”的目标。

这一年是狠抓生态文明、促进绿色持续发展的一年。

环境保护不断加强。一是深入开展“蓝天”保卫战。相继开展城市建成区禁放烟花爆竹、农村秸秆禁烧、燃煤锅炉整治、关停取缔砖瓦黏土企业、“地条钢”非法生产、餐饮油烟、建筑扬尘等专项治理，关闭“小散乱污”企业13家，责令整改197家。空气质量显著改善，城区空气PM2.5和PM10日平均浓度均较2017年降低10个百分点以上，空气优良天数增加31天，优良率达93%。二是深入开展“碧水”保卫战。开展城区饮用水水源地环境问题专项整治，集中开展水库水质整治和畜禽养殖污染防治。全区214家小(二)型以上水库签订退出承包养殖合同，实现“人放天养”，关闭禁养区内规模化养殖场78家。启动生活污水处理厂和抚北工业污水处理厂扩容提标改造前期工作，完成污水管网建设3.2公里。城区集中式饮用水水源地水质达标率100%，地表水出境考核断面水质稳定在Ⅲ类以上。三是深入开展“净土”保卫战。加强固体废物规范化管理，规范危险废物收集、储存、处置行为。加快推进抚北工业园区下游土壤综合治理修复项目和农业土壤修复试点项目。

生态创建不断加快。深入开展严厉打击破坏森林资源涉林违法犯罪专项行动，继续实施退耕还林、封山育林、天然林保护等工程，打造山清水秀的生态环境。截至目前，我区森林覆盖率达到46.3%。投资7.1亿元的抚河流域生态保护及综合治理PPP项目一期工程稳步推进。22座小(二)型水库改造工程、千金坡倒虹吸管改造工程已全面完成。基本完成了在我区辖区内廖坊水利枢纽灌区二期干渠主体工程，到2019年基本实现春灌目标。

体制机制不断完善。制定《临川区领导干部自然资源资产离任审计暂行办法》，持之以恒抓好“河长制”“湖长制”“林长制”“路长制”的贯彻落实，构建区、乡、村、组四级责任体系。严格落实生态保护红线制度，完善生态红线管制措施，加强耕地和基本农田保护。完善生态建设考核制度，实施有区别、有侧重的绩效评价考核，将生态文明建设纳入乡镇目标管理考评。严格执行抚河流域治理、公益林生态补偿等政策，鼓励和引导社会资本广泛参与生态文明建设。建立环境执法联动机制，对重点污染源企业落实在线监控管理制度。

这一年是狠抓民生民本、促进百姓福祉增进的一年。

脱贫攻坚亮点纷呈。深入开展“春季攻势”“夏季整改”“秋冬会战”专项行动，扎实推进脱贫攻坚工作。在教育扶贫方面，创新建立教师、帮扶干部双结对联动机制和区、乡、校三级对接工作机制，确保贫困家庭子女教育扶持全覆盖。在健康扶贫方面，全面落实“先诊疗、后付费”“一站式服务”“五道保障线”等措施，推行家庭医生与帮扶干部双结对联动机制，定期开展送医下乡、免费体检等健康扶贫活动，最大程度减少因病致贫返贫。在安居扶贫方面，完成建档立卡贫困户危旧房改造1233户，确保贫困户住房安全有保障。在产业扶贫方面，积极探索“五定三建一受益”模式，进一步调整和优化产业全覆盖实施方案，让贫困群众深度融入产业发展。在基础设施扶贫方面，高标准制定龙溪镇汤家村、高坪镇东源村、荣山镇新安村、湖南乡山塘村等4个村的村庄整治计划，按步骤分期分批落实扶贫项目。2018年全区减贫1521人，贫困人口减少到5369人，贫困发生率下降到0.75%，最后一批4个贫困村将全部退出。

民生温度显著提升。坚持新增财力向民生倾斜，教育、医疗、社保等民生支出达49.02亿元，增长17.2%，50件民生实事全面完成。临川第一实验学校二期工程全面竣工，全区23个新建改建“一乡一园”工程全部开工，重点推进临川二小新校区、河西学校和第一保育院等重点校建项目。新引进海南昌茂教育集团，成功盘活原美佛儿学校，重塑民校品牌。加快构建覆盖城乡的卫生计生和健康服务体系，启动国家健康促进区和省级慢性病综合防控示范区创建工作，重点推进区第一人民医院新院区、区中医院新院区等一批卫生重点项目建设，完善和拓展临川区智慧百乡千村健康医养扶贫工程各项功能。扎实推进国家历史文化名城创建工作，全区12个村落成功入选“中国传统村落”，白浒窑陶瓷非遗项目赴英国剑桥大学参加国际艺术展示，配合市委、市政府成功举办汤显祖戏剧节暨国际戏剧交流月活动。

社会大局和谐稳定。深化平安临川建设，积极探索“人在网中走、事在格中办”网格化新型社会治理体系，扫黑除恶专项斗争取得阶段性成效，共立涉黑案件1起，打掉恶势力犯罪集团8个、恶势力犯罪团伙6个，全区信访形势总体平稳可控、不断向好，荣获全省信访工作“三无县”，公众安全感和群众满意度进一步提升。严格落实安全生产“党政同责、一岗双责”，烟花爆竹生产企业全面退出，深入开展“扫雷”“清零”行动，确保全区安全生产平稳态势。深入开展全领域食品安全专项整治月行动，严厉打击各种食品安全违法行为。加大打击非法集资力度，开展地方政府债务清理清查和问题整改工作，有效防控金融风险。

这一年是狠抓改进作风、促进自身建设加强的一年。

以建设“五型”政府为契机，坚决把党的政治建设放在首位，坚决整治政府系统“怕、慢、假、庸、散”等作风顽疾，特别是大力整治怕作为、慢作为、不作为等作风顽疾，优化办事流程，提高办事效率。抓好红色基因教育，广泛开展诵读《红色家书》活动。深化“七五”普法教育，重点推进一户一位“法律明白人”培养工程，推动全区农(居)民形成“办事依法、遇事找法、解决问题用法、化解矛盾靠法”的习惯。加强党风廉政建设，加大力度整治群众身边不正之风和腐败问题，开展扶贫领域腐败和作风问题专项治理，政府形象持续提升。自觉接受区人大及其常委会法律监督和工作监督、政协民主监督、社会监督和舆论监督，办理人大代表建议44件、政协委员提案127件，办结率达100%；收到区长热线群众诉求1835件，书面登记受理661件，办结率达100%。

同时，武装工作扎实有效，顺利完成民兵调整改革和兵员征集任务，扎实开展军事训练，国防动员、民兵预备役建设、人民防空、优抚安置和“双拥”共建等工作都取得新的成绩。扎实推进退役士兵信息采集工作，维护军人军属合法权益。民族宗教、外事侨务、涉台事务、应急管理、统计调查、防震减灾、气象、档案、地方志、妇女儿童、老龄、残联、科协、红十字会等工作有了新的提升。

各位代表，过去的一年，一路高歌猛进，我们奋力拼搏；怀揣激情梦想，我们再创辉煌。这是市委、市政府和区委坚强领导的结果，是区人大、区政协和社会各界监督支持的结果，是全区人民同心同德、团结拼搏的结果。在此，我谨代表区人民政府，向全区广大干部群众，向人大代表、政协委员、各民主党派、工商联、无党派人士、人民团体及社会各界人士，向各驻区单位、驻区部队官兵，向为临川发展积极献计出力的老领导、老同志，向外来投资者和建设者，向所有关心、支持和帮助临川发展的同志们、朋友们，表示衷心的感谢和崇高的敬意!

我们也清醒地认识到，经济社会发展中不平衡、不充分的问题依然存在，主要表现在：经济下行压力加大，产业结构调整正处于新旧动能转换期，工业支撑作用还有待提升；基础设施建设滞后于城市的快速发展，一些城市病问题有待解决；创新基础相对薄弱，创新型、开放型人才相对不足；资源环境约束加剧，生态文明建设任务依然繁重；社会治理和公共服务事业存在短板；少数干部思想保守、担当意识不强，离“五型”政府建设要求还有待进一步提高等等。对此，我们一定认真对待、绝不回避，以担当实干的态度和气魄，切实加以解决，绝不辜负全区人民的期望!

二、2019年工作安排

2019年是新中国成立70周年，是决胜全面建成小康社会第一个百年奋斗目标的关键之年，扎实做好全年各项工作责任重大，意义深远。今年政府工作的总体要求是:始终坚持以习近平新时代中国特色社会主义思想为指引，深入贯彻中央经济工作会议和省委十四届七次全会、市委四届六次全会、区委四届六次全会精神，贯彻新发展理念，落实高质量发展要求，接续奋斗“转作风、做民事、促稳定、保发展”工作总部署，撸起袖子加油干、扑下身子抓落实，全面决战工业崛起、城市提质、乡村振兴、全面小康、作风转变，吹响进军“领头羊”位置集结号，书写临川发展史上崭新的时代篇章。

今年经济社会发展的主要预期目标是:完成生产总值增长8.7%，力争增长9%；财政总收入增长5%，其中一般公共预算收入增长3%；规模以上工业增加值增长9.5%，力争增长9.8%；固定资产投资增长10%，力争增长11%；社会消费品零售总额增长9.5%，力争增长10.5%；实际利用外资增长10%；出口总值增长1.2%；城镇居民人均可支配收入增长8.5%，农村居民人均可支配收入增长9%。

围绕上述目标要求，重点做到“六个坚持”:

(一)坚持“发展为先”。

坚持以更大勇气、更大力度全面深化改革，全面释放改革红利，积极拓展对外开放广度和深度，开启扩大开放融合新征程。

以改革促发展。稳步推进机构改革有关工作，科学设置部门和内设机构，在1月底前基本完成机构调整工作，在3月底前全面完成改革任务，做到思想不乱、工作不断、队伍不散、干劲不减。稳步推进“放管服”改革，力争政务服务事项“一次不跑”“只跑一次”办理率、网上可办率、“一窗受理、集成服务”受理率均达70%以上，与日常生产生活密切相关事项的办理窗口实行全天候服务，着力打造24小时“不打烊”的在线服务大厅。稳步推进农村集体产权制度改革和农村宅基地管理试点工作，进一步促进农村各类资源优化配置、盘活农村集体资产。同时，继续稳妥推进医疗卫生教育等社会事业、城乡融合发展、文化事业和公共文化服务、绿色殡葬、社会治理体制、财税金融体制和生态文明体制等重点改革事项。

以融合促发展。一方面，加快融入南昌。以打造南昌大都市圈为契机，重点推进昌抚合作示范区建设，做好启动区控制性详细规划，围绕现代农业、文化教育、休闲旅游、养生养老、基础设施等方面谋划一批重大项目。积极推进修建供水工程、云山110KV输变电工程、福银高速互通等重点基础设施项目和昌抚家和特色小镇项目前期工作。对续建的昌抚态何源田园综合体、昌抚牡丹田园综合体项目倒排工期，加快建设进度，确保早竣工、早投产、早见效。另一方面，加快融入中心城区。开展“三城同创”和城市功能与品质提升三年行动，做到与市中心城区同档同质提升。抓紧抓实治脏、治乱、治堵、功能修补、生态修复、特色彰显、亮化美化、治理创新“八大行动”，坚决杜绝“两违”行为，重点解决群众最关心的城市问题，力争城市功能与品质“一年有提升，两年上台阶，三年进一流”。

以开放促发展。抓住国家扩大开放、扩大进口有利时机，在鼓励原有的企业扩大出口规模和提升效益的基础上，着力引进培育一批有规模的生产型出口企业，力争新增注册和变更外资企业7家。强力推动“两转”工作，实施出口市场多元化战略，着重培育区域内高附加值产品开拓市场。力争全年实现外贸出口15.9亿元，实际利用境外资金5750万美元，其中完成现汇进资1200万美元。继续鼓励推动有实力的企业参与国际产能合作，设立营销网点，开拓新兴市场，带动产品出口，力争中欧货运班列按季度常态化开行，努力让每一趟班列“满载而出”“满意而归”。

(二)坚持“项目为王”。

牢固树立“不抓项目是失职、不会抓项目是不称职”的导向，以项目建设稳投资促增长、提质量增后劲、论英雄定奖惩。

狠抓项目建设。2019年，初步筛选重点项目55个、总投资296亿元。按城市主干道标准对抚八线进行提升改造，第一期将抚八线才都工业区段改造实施好。加快“一河两岸”工程建设进度，消除上顿渡城区与才都工业区之间空间壁垒，将才都工业区、“一河两岸”融为一体，真正实现产城融合。积极谋划电子科技产业园二期、临川人才公寓等重点项目，继续抓好才子大桥、城区雨污管网改造工程、平安大道综合地下管廊工程、第二水厂等一批重点城建项目建设，不断完善城区功能。

狠抓招商引资。全面开展“三请三回”和“三企”入赣活动，继续实行区委、人大、政府、政协四大招商兵团和四个专业驻点招商小分队，围绕电子、食品、医药三大主导产业，开展精准招商、以商招商、产业招商，着力引进综合实力突出的“旗舰型”企业、竞争力强的行业“领头羊”、细分领域“隐形冠军”和高科技“独角兽”企业。力争2019年新引进投资亿元以上重大项目24个。加快产业集群发展,力争电子科技产业园全面入驻并建成投产，实现税收4亿元以上。积极推进大健康食品产业园与良品铺子、三只松鼠等知名食品企业合作落地，新建30万平方米厂房，力争年底项目建成试投产。加快推进宝芝林生物医药产业园项目建设。

狠抓跑项争资。牢牢把握国家产业政策和投资导向，紧紧抓住省委、省政府构建“一圈、两轴、三区”区域发展新格局、省生态文明先行示范区、鄱阳湖生态经济区及抚州全面深化区域合作加快发展等重大机遇，谋划储备项目，锲而不舍地申报争取项目，做实做优重大项目储备库，形成“储备一批、申报一批、开工一批”梯次推进格局，力争2019年有一批带动性强、成长性好的项目挤进国家和省规划“笼子”，向上争取各类资金22亿元。

(三)坚持“产业为基”。

坚持以产业为基础、为支撑的高质量发展，加快构建链条完整的现代化产业体系，大力发展新经济，不断提升产业层次、结构、规模和效益。

挺起工业脊梁骨。一是推动骨干企业做大做强。围绕“众创业、个转企、小升规、规转股、股上市、强龙头、育集群”的企业成长主线,落实好“降成本、优环境”的各项政策，扩大“财园信贷通”“银税互动”等规模，打好企业梯次培育组合拳。扶强龙头企业，重点加快生产要素、优质资源的集聚，加快企业上市步伐，支持其裂变扩张、唱响品牌。扶大规上企业，鼓励其技改扩建、科技创新。扶优中小企业，全面落实扶持中小企业创业发展的各项政策，通过“父母式”服务，确保每年培育10户以上企业入规。二是推动传统产业升级转型。围绕电子信息、食品加工、生物医药、有色金属加工等主导产业实施大配套，借助电子科技产业园先进的运作模式引进半导体、先进通讯设备、新材料等现代高新电子信息产业项目，力争全年园区新增落户亿元以上项目16家，新增投产企业8家。三是推动工业园区提质扩容。大力实施“两型三化”管理提标提档行动，树立“以亩产论英雄”导向，力争抚北工业园区主营业务收入突破200亿元大关、税收突破10亿元大关。全面提升抚北工业区，重点抓好工业大道以北延伸段道路以及供水、电网、污水处理厂尾水排放等配套工程建设，进一步完善社会功能。加快“腾笼换鸟”，确保2020年底“僵尸企业”全部处置到位。强力推进才都工业区，力争新增用地2000亩，完成东一环及工业大道东段道路建设，启动东纵二路、西一环南延伸北段等道路建设。

奏响乡村振兴曲。突出产业兴旺，着力调优农业产业结构，充分发挥昌抚牡丹田园综合体、昌抚态何源田园综合体、华宸农业、仙盖山等综合体辐射带动作用，重点培育和壮大龙鑫生态、民生科技等一批农业产业化企业，加快实现传统农业向现代农业转型升级。加快发展以中药材、生态水果、绿色出口蔬菜为重点的特色种植业，以生态畜禽、特色水产为重点的特种养殖业，着力构建“一乡一特”、形成“一村一品”。与此同时，大力推进“中国好粮油”行动示范区建设，提升粮食品质，力争粮食播种面积稳定在133万亩以上,粮食总产稳定在64万吨以上。突出生态宜居，重点抓好600个新农村建设村点“七改三网”基础设施建设和“8+4”公共服务配套建设任务，保持乡村特色、留住乡愁乡韵。深化农村人居环境整治三年行动，以“干净、整洁、有序”为基本要求，以“拆得彻底、清得干净、改得美观、管得规范”为目标，加快推进环境卫生整治全域覆盖，全面提升农村环境品质。突出乡风文明，推动移风易俗，树立文明乡风，共同凝聚向上、崇善、爱美的强大正能量。突出治理有效，坚持自治、法治与德治相结合，发挥乡规民约的作用，确保农村社会和谐稳定。突出生活富裕，狠抓产业富农和创业富农，拓宽农民增收渠道，让农民的口袋鼓起来。加快发展村级集体经济，力争85%以上的村集体经济都达到5万元以上。

打好三产融合牌。做好“旅游+”文章，围绕发展全域旅游，抓住国家促进乡村旅游发展提质升级有利时机，大力发展以特色种养、乡土风情、地域文化、林业生态、水生态、古村落为重点的乡村休闲旅游，重点抓好仙盖山省5A级乡村旅游示范点二期工程建设，力争昌抚牡丹田园综合体创省4A级乡村旅游示范点。推动“旅游+农业”“旅游+教育”“旅游+健康”“旅游+餐饮”的发展，协调不同产业之间的共同发展，实现旅游产业升级。做好“互联网+”文章，积极推进国家级数字农业示范县(区)建设，促进互联网、云计算、大数据技术与现代制造业、现代农业和现代服务业的融合。大力发展农村电子商务，着力培育一批电商村、特色电商涉农产业和龙头企业，使其成为带动农民增收致富的新途径。做好“健康+”文章，着力构建多层次养老服务体系，提升养老服务供给水平；支持昌抚合作示范区重点发展健康养生养老产业，积极推进家和特色小镇等重大项目。做好“金融+”文章，加快引进银行业金融机构步伐，力争新引进2家银行业金融机构。适时向社会推介PPP项目，落实各项激发民间投资活力政策措施，鼓励和引导社会资本参与到项目建设中来。

(四)坚持“生态为重”。

加强生态文明建设，以山为骨、以水为脉、以林为衣，精心雕琢勾勒临川生态画卷，把临川的美充分展示出来、释放出来。

注重生态建养。着力加强生态修复与建设，深入实施“净空、净水、净土”专项行动，让群众生活在天更蓝、山更绿、水更清的优美环境之中，深入实施封山育林等生态保护工程，确保全区森林覆盖率稳定在46.4%以上。加强水库水质环境整治，严厉打击河道非法采砂行为，全力保护水生态，全面打造河长制升级版。加快构建低碳循环的绿色工业体系、生态有机的绿色农业体系、集约高效的绿色服务业体系。

注重环境监管。坚决打好污染防治攻坚战，严格按照时序进度稳步推进中央环保督察反馈问题、长江经济带生态环保审计问题、长江经济带“共抓大保护”执法检查问题的整改落实，全面完成禁养区内规模养殖场搬迁、拆除工作，限养区、可养区内完成养殖设施整改或搬迁。加强重点排污企业监管，确保污染物排放稳定达标。继续保持高压态势，坚决打击各类环境违法行为，对拒不整改、恶意排污的企业，实行“顶格处罚”。

注重长效机制。按照“党政同责、一岗双责、权责一致、齐抓共管”和“谁主管谁负责、谁决策谁负责”的原则，严格落实“管发展必须管环保、管行业必须管环保、管生产必须管环保”的生态环境保护工作责任体系及问责制度。加快打通绿水青山和金山银山的双向转换通道，探索建立公益林补偿标准动态调整机制和湿地的生态补偿制度，逐步提高补偿标准，形成科学合理的生态效益补偿标准体系。

(五)坚持“民生为本”。

始终把让群众过上更好生活作为奋斗目标，提高和改善民生水平，加强和创新社会治理，使人民获得感、幸福感、安全感更加充实、更有保障、更可持续。

全力推进脱贫攻坚。坚决打赢脱贫攻坚战,大力推进脱贫攻坚“十大工程”，着力巩固脱贫成果，逐步建立稳定脱贫长效机制，确保帮扶力度不减、支持政策不变、脱贫成效不反弹，确保4342名贫困人口如期脱贫。同时，有序地推动城镇贫困群众脱贫解困工作，使城镇困难群众达到“两不愁、三保障”目标，确保全面小康路上农村贫困人口和城镇贫困群众一个都不能少。

持续完善社会保障。把稳就业摆在更加突出的位置，加大稳岗支持力度，建立健全创业资金连续帮扶机制，推行终身职业技能培训制度。加快健全覆盖城乡的社会保障体系，稳步提高城乡基本养老、基本医保、城乡低保等补助标准，推进基本医保跨省异地就医住院费用直接结算。完善社会救助制度，统筹推进扶老、助残、救孤、济困等社会事业发展。坚持房地产市场调控目标不动摇，继续实施保障性住房安居工程，扎实推进棚户区和农村危旧房改造。

协调发展社会事业。紧盯群众的痛点、难点和堵点，集中力量抓好普惠性、基础性、兜底性民生建设，切实解决好教育、医疗等实际问题，重点推进临川二小新校区、河西学校、文昌里九年一贯制学校、区第一保育院、区第一人民医院新院区、区中医院新院区等民生社会类重点项目。进一步促进城乡教育均衡发展，支持临川实验学校等民办学校发展，加快推进乡镇公办中心幼儿园扫尾工作。进一步实施文化强区战略，加快临汝书院、临川文化中心建设，着重打造乡镇(街道)和村级(社区)综合文化服务中心，加大文物和传统村落保护力度，重视文化的培育、文风的塑造和文脉的传承。举办好农民丰收节和临川首届教育文化艺术节活动，推动物质文明和精神文明协调发展。坚持不懈推进“厕所革命”，努力补齐影响群众生活品质的短板。

加强创新社会治理。加强信访积案化解和源头防范，对各种矛盾纠纷做到早排查、早发现、早稳控、早处理。深入推进扫黑除恶专项斗争，发现一起、查处一起，坚持露头就打、除恶务尽。推进安全生产领域改革发展，扎实推进企业安全生产标准化和安全风险管控试点示范建设。强化食品药品重点领域专项治理，抓好重点产品质量监管，保持执法打假高压态势。加强国防动员和后备力量建设，深化军民融合，做好人民防空和拥军优抚安置工作。依法严厉打击非法集资等违法金融活动，坚决守住不发生系统性、区域性金融风险的底线。

(六)坚持“实干为要”。

各位代表，发展的重任、竞争的态势、人民的期盼，对政府建设提出了更高要求。我们将坚持用习近平新时代中国特色社会主义思想武装头脑，“实”字当头，“干”字为先，全力打造忠诚型、创新型、担当型、服务型、过硬型政府。

打造忠诚型政府。树牢“四个意识”、坚定“四个自信”，坚决做到“两个维护”，自觉在思想上、政治上、行动上同以习近平同志为核心的党中央保持高度一致，强化政治责任，保持政治定力，把准政治方向，提高政治能力，严守政治纪律和政治规矩。

打造创新型政府。全面启动全省创新型县(区)创建工作，深入推进创新驱动发展战略，重点聚焦电子信息、生物医药、食品加工、有色金属加工等主导产业，着力深化科技体制机制改革，大力推动科技成果转移转化，加快建成国家级有色金属和医药食品领域的工程技术研究中心。

打造担当型政府。把功夫下在狠抓工作落实上，对政府各项重点工作制定严格的“时间表”，推动任务目标化、目标项目化、项目责任化、责任实效化。大力推行“一线工作法”，做到问题在一线发现、矛盾在一线解决，努力打通政策落实的“最后一公里”。

打造服务型政府。坚持以问题为导向，以“群众办事百项堵点”为靶心，紧紧围绕服务企业全生命周期、深化“放管服”改革、“互联网+政务服务”和“降成本优环境”专项行动等建设服务型政府的重点工作，想群众和企业之所想，急群众和企业之所急，在提升服务效能中展示政府新面貌。

打造过硬型政府。切实加强法治政府建设，带头学法懂法守法用法，真正用法律管好政府这只“有形之手”，使临川高质量、跨越式发展始终在法治轨道上行稳致远。坚持从源头上减权限权、规范用权，让清正清廉清明成为全区政府系统和全体政府工作人员的鲜明底色。

各位代表，让我们更加紧密地团结在以习近平同志为核心的党中央周围，以习近平新时代中国特色社会主义思想为引领，在市委、市政府和区委的坚强领导下，以咬定青山的决心、快马加鞭的姿态、争创一流的势头、担当实干的作风干出一番新业绩、闯出一片新天地，为建设美丽幸福大临川不懈奋斗!
